# A Multidimensional Benchmark of Public EEG Datasets for Driver State Monitoring in Brain–Computer Interfaces

**DOI:** 10.3390/s25247426

**Published:** 2025-12-06

**Authors:** Sirine Ammar, Nesrine Triki, Mohamed Karray, Mohamed Ksantini

**Affiliations:** 1 Advanced Technologies for Image and Signal Processing (ATISP) Lab, École Nationale d’Électronique et des Télécommunications de Sfax, University of Sfax, Sfax 3018, Tunisia; sirine.ammar@enetcom.u-sfax.tn (S.A.); mohamed.ksantini@ipeis.usf.tn (M.K.); 2 ESME Research Lab, ESME, 94200 Ivry sur Seine, France; 3 Control and Energy Management (CEM) Laboratory, École Nationale d’Ingénieurs de Sfax, University of Sfax, Sfax 3038, Tunisia; nesrine.triki@isims.usf.tn

**Keywords:** BCI, EEG, driving simulation, cognitive load, emotion recognition, driver monitoring, public datasets, multimodal signals, intelligent transportation systems, machine learning, deep learning

## Abstract

Electroencephalography (EEG)-based brain-computer interfaces (BCIs) hold significant potential for enhancing driver safety through real-time monitoring of cognitive and affective states. However, the development of reliable BCI systems for Advanced Driver Assistance Systems (ADAS) depends on the availability of high-quality, publicly accessible EEG datasets collected during driving tasks. Existing datasets lack standardized parameters and contain demographic biases, which undermine their reliability and prevent the development of robust systems. This study presents a multidimensional benchmark analysis of seven publicly available EEG driving datasets. We compare these datasets across multiple dimensions, including task design, modality integration, demographic representation, accessibility, and reported model performance. This benchmark synthesizes existing literature without conducting new experiments. Our analysis reveals critical gaps, including significant age and gender biases, overreliance on simulated environments, insufficient affective monitoring, and restricted data accessibility. These limitations hinder real-world applicability and reduce ADAS performance. To address these gaps and facilitate the development of generalizable BCI systems, this study provides a structured, quantitative benchmark analysis of publicly available driving EEG datasets, suggesting criteria and recommendations for future dataset design and use. Additionally, we emphasize the need for balanced participant distributions, standardized emotional annotation, and open data practices.

## 1. Introduction

**Figure 1:**
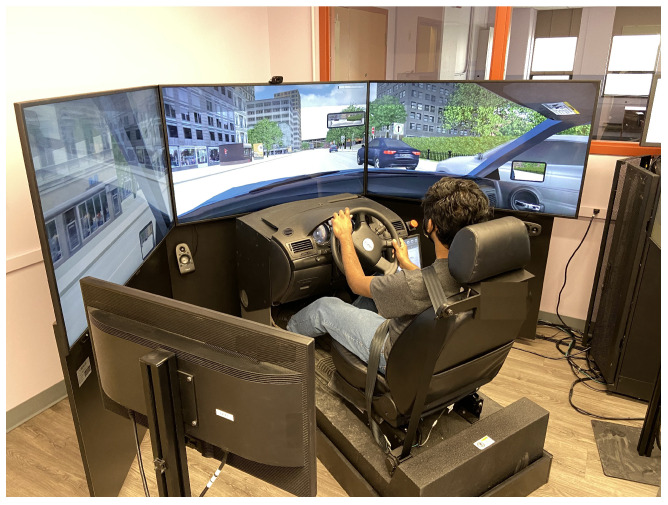
EEG-based BCI setup for monitoring driver cognitive states during simulated driving [1].

As a primary sensor modality in intelligent transportation systems, electroencephalography (EEG)-based brain–computer interfaces (BCIs) have been deployed for real-time measurement of driver states, including drowsiness (or reduced alertness due to fatigue); cognitive workload (or mental demands to perform driving tasks); and affective states (such as stress or frustration). This capability enables adaptive vehicle technologies to dynamically compensate for driver status, thereby reducing accidents and enhancing safety. However, EEG technologies remain constrained by the need for relevant EEG data collected in vehicular contexts that emulate real-world driving tasks, such as simulated environments or real-road testing environments, as shown in Figure 1.

Some researchers have made it possible for others in the domain to use their data as public data following public policies on sharing data (using public open access repositories), while others have chosen a closed approach to their data, making such datasets inaccessible. Many studies have examined cognitive states in driving, but their contributions have often been limited by issues such as restricted data access, practical utility of datasets, and methodological inconsistencies. For instance, ref. [2] specifically investigated working memory during driving, using electroencephalogram (EEG) microstate decomposition as a measure of the transient global network configurations in the brain. Their data, collected in a high-fidelity driving simulator, involved both arithmetic and phone-use tasks, and they identified EEG microstates as outperforming spectral power models with respect to not only differentiating cognitive load intensity but also predicting driver behavior. However, the authors did not provide access information for their dataset, limiting opportunities for secondary analysis and follow-up research. Similarly, ref. [3] created a fatigue detection system based on EEG collected during driving; however, this dataset is also unavailable to the research community, impeding continuity. Also, the authors in [4] analyzed EEG data during real-world urban driving (not simulated), to investigate cognitive workloads, yet the dataset was only described in the publication without open accessibility. In addition, the SEED-VIG dataset [5] has become a widely used benchmark, but limited to simulated driving videos in prerecorded footage rather than interactive scenarios, lacking ecological validity (realism for real-world application) for affective state recognition. These studies reveal limitations including unavailable public datasets, the use of non-interactive or simulated protocols and inconsistent labeling and documentation standards. Also, the field suffers from divergence in task realism (how closely tasks mimic real driving), signal modalities (types of data recorded, EEG, eye-tracking, etc.), annotation quality (accuracy of labels for states like “drowsy”), and licensing policies. These gaps undermine research progress and prevent the development of generalizable model systems that perform consistently across diverse scenarios suitable for deployment in reliable ADAS. To address this issue, this study provides, to the best of our knowledge, the first structured and quantitative benchmark analysis of publicly available EEG datasets relevant to vehicle-driving contexts. The benchmarking process involved systematic evaluation of seven datasets (SEED-VIG, CL-Drive, MPDB, Sustained-Attention, VMI-BCI, PPB-Emo and Emergency Braking) based on multiple criteria, including task design realism, multimodal integration (such as Electrodermal Activity (EDA) and eye-tracking), labeling granularity, and accessibility constraints. We also investigate model performance trends, with a particular focus on multimodal fusion techniques. Our systematic evaluation follows PRISMA (Preferred Reporting Items for Systematic Reviews and Meta-Analyses) standards [6]. The remainder of this paper is structured as follows: Section 2 presents the methodology, which includes the PRISMA systematic review protocol, dataset selection criteria, and metadata extraction process. Section 3 presents results comparing dataset characteristics and model performance trends. Section 4 discusses gaps related to demographic diversity, ecological validity, and affective monitoring, offering suggestions for improvement. Lastly, important conclusions and translational implications are summarized in the final section.

## 2. Methods

For methodological rigor, this systematic review adheres to the Preferred Reporting Items for Systematic Reviews (PRISMA) guidelines; the completed PRISMA checklist is provided as Supplementary Material. PRISMA offers an evidence-based checklist to address deficiencies in reporting in systematic reviews, thereby enhancing replicability and scientific rigor [6]. Following PRISMA guidelines, we developed a protocol describing dataset selection criteria, search strategy, metadata extraction methods, and analysis processes. This study was conducted in accordance with a preregistered protocol available on the Open Science Framework (OSF): https://doi.org/10.17605/OSF.IO/GXSZT (accessed on 6 November 2025).

**Figure 2:**
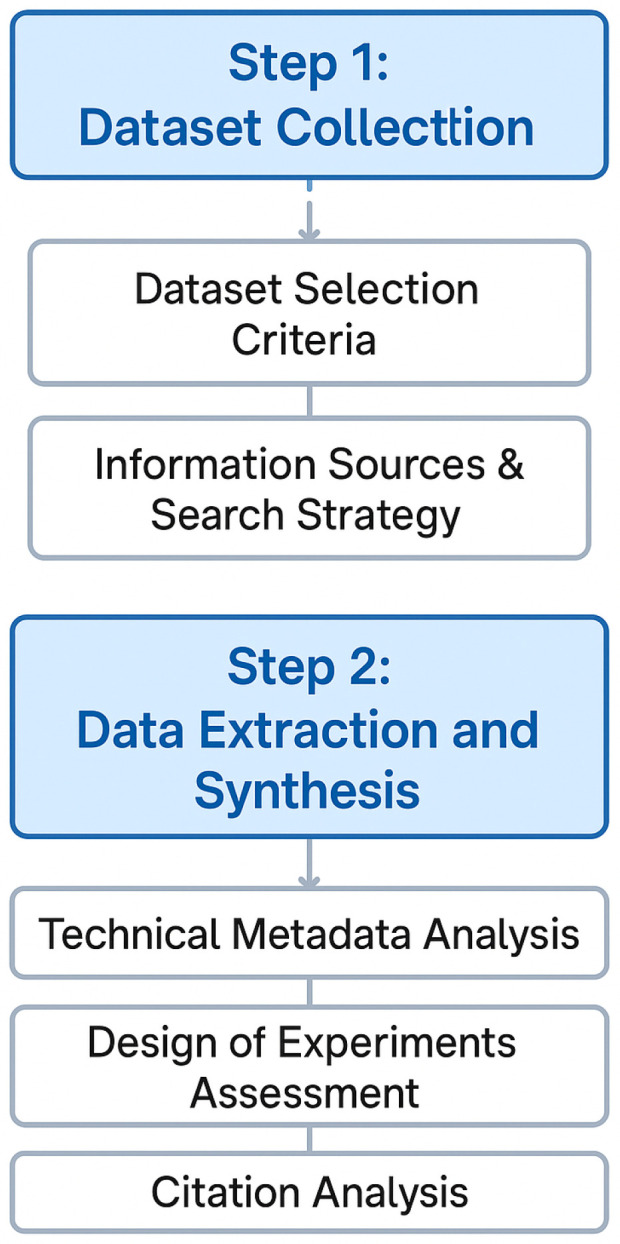
Systematic review workflow.

This descriptive benchmarking study analyzes and synthesizes characteristics of datasets and performance measures that have been reported in previous studies. Therefore, no new data were collected through model retraining or reprocessing of existing experiments. Figure 2 illustrates the two-phase structured approach. The process begins with the Dataset Collection phase, which is conducted via a systematic search strategy across various information sources and based on established selection criteria. The subsequent data extraction and synthesis phase follows. It comprises three separate analytical modules: technical metadata analysis (visualizing key dataset characteristics), experimental design assessment (identifying missing or underrepresented aspects), and citation analysis (supporting cross-dataset validation and evidence-based benchmarking).

### 2.1. Dataset Collection

The original selection of datasets is made based on three guiding principles: the dataset uses EEG signals, involves driving-related scenarios, and is publicly accessible. The search identified 13 datasets. After going through a further iterative selection process, 7 datasets are identified as candidates for inclusion.

#### 2.1.1. Dataset Selection Criteria

- EEG modality: The inclusion of EEG signals as the main measurement modality. This is due to their unique ability to directly and temporally characterize neural dynamics, which will be essential for understanding driving states from a more authentic perspective [4].
- Driving-related tasks: Tasks related to driving, whether real-world execution, high-fidelity simulations, or neurocognitive imagination paradigms, must maintain realism and practical applicability. This specific criterion places research in real driving situations, such as assessing responses to hazards in a simulator or mental workload when planning a route-on-road [7,8].
- Public accessibility: Making public datasets available allows for independent verification, algorithm benchmarking, and community-based improvement. This guarantees future research based upon open data, which is critical for cumulative scientific progress in EEG-based driver monitoring.

Thirteen datasets are primarily identified using these three criteria: EEG Driver Fatigue Detection [9], Sleepy Driver EEG Brainwave Data [10], EEG Dataset Recorded In A Car Simulator [11], Multimodal Cognitive Load Classification Dataset [12], Cognitive load during driving dataset [2], Driving Physiological and Vehicle Data Multimodal Fusion Dataset (DPV-MFD) [13], CL-Drive [1], MPDB [14], Sustained-Attention [15], Emergency Braking [16], VMI-BCI [17], SEED-VIG [5], and PPB-Emo [18]. A complete list of excluded datasets with specific justifications is provided in Table A1 in Appendix A. To continue refining the benchmarking process, and ensure to only analyze resources that are the strongest and most impactful, three additional refinements are made. These refinements add more selectivity, removing datasets that have no peer-reviewed validation, do not provide full metadata for replicability, and have not been recently collected in the last 5–6 years for consistency with contemporary research standards and technologies.

- Peer-reviewed publication: Datasets must come from peer-reviewed articles, not other types of non-validated data (such as Kaggle submissions), and should ensure the academic and methodological integrity and credibility of the underlying data. Complete metadata is required, such as experimental protocols, participant characteristics (age, gender, etc.) and task parameters, to provide vital context for understanding outcomes and alignment across studies.
- Recent collection: Collections that are older than modern EEG/BCI technologies (generally pre-2016) are excluded to focus on collections that align with current signal processing and machine learning models.
- Elevated participant thresholds: Collections with narrow sample datasets (number of subjects <10) are rejected since the low statistical power does not meet the required inter-subject variability and statistical power that are needed for generalizable model development [19,20]. At the same time, strict quality standards require documentation on the applied signal preprocessing, artifact removal, and annotation reliability to assess the integrity and replicability of the analysis.

In total, this comprehensive search yielded seven datasets: CL-Drive, MPDB, Sustained-Attention, Emergency Braking, VMI-BCI, SEED-VIG, and PPB-Emo.

#### 2.1.2. Information Sources and Search Strategy

The comprehensive literature and dataset search was conducted across many information sources, including IEEE Xplore, ScienceDirect, arXiv, FigShare, Kaggle, GitHub, Google Scholar, and PhysioNet. The key search terms were “EEG”, “driving”, “dataset”, “brain-computer interface”, and “drowsiness detection”. The search included peer-reviewed articles and datasets publicly accessible online.

### 2.2. Data Extraction and Synthesis

Once the datasets are collected, technical and experimental metadata are extracted from each dataset and summarized in Table 1. This includes the number of subjects, the EEG channels, the type of task and the labeling scheme. Furthermore, all machine learning/deep learning models with performance metrics obtained from the citation analysis are recorded. These performance measures are all reported in the literature and are not evaluated against a reference standard. This study synthesizes the reported metadata and performance measures without reevaluating the raw signals. The result is an inability to directly compare and contrast across datasets, particularly for multimodal fusion results, due to technical differences in data collection (sensor calibration, time-synchronization across modalities). These differences are inherent to the original studies, hindering standardization in this analysis.

Table 1 summarizes the collected metadata of the analyzed datasets. Major characteristics include acquisition parameters, subject demographics and accessibility levels.

To develop a formalized openness scoring system, datasets are assigned an openness score based on licensing and accessibility conditions:

- ★★★ High Openness: Datasets that are publicly available via licenses like Creative Commons Attribution 4.0 (CC BY 4.0) and are downloadable on GitHub or Figshare as examples.
- ★★ Moderate Openness: Datasets that are available on request from the authors or stored under formal contracts that allow them to be shared in an academic context.
- ★ Low Openness: Datasets that are not available to the public or have restricted or vague licensing conditions. These datasets were not considered in this study for purposes of ensuring openness, continued access for research, and alignment with open scientific practice.

#### 2.2.1. Multidimensional Scoring Framework

To enable structured comparative evaluation and support analytical rigor, a comprehensive scoring system was designed. This system uses a standardized 0–5 scale, across six key dimensions, to review the fundamental aspects of dataset quality for driver monitoring applications. The proposed system also incorporates weighted composite scores to reflect each dimension’s relative importance for deployment in the real world. Dimension-specific criteria listed in Table 2 are the following:

Demographic Diversity (0.20 weight) contrasts the age span, for example wide (>40 years) versus narrow (<10 years), and gender balance, for example balanced (45 to 55%) versus severe bias (>80% one gender).

Ecological Validity (0.25 weight) favors real-road driving versus simulated scenarios.

Modality Richness (0.15 weight) assesses additional auxiliary signals beyond EEG.

Annotation Quality (0.15 weight) distinguishes multi-modal continuous labeling versus basic task labels.

Accessibility (0.10 weight) varies from CC-BY direct download to restricted access.

Technical Quality (0.15 weight) considers whether documentation is complete and sample size is sufficient (to score full points, the dataset should cover more than 30 subjects with complete documentation).

Weight assignments (Wt in Table 2) account for the priority of domains, with ecological validity and demographic diversity being the most important as they have the most impact on real-world applicability and fairness of the model.

Composite scores are expressed as $S_{composite}=\sum_{i=1}^{6}w_{i}\times s_{i}$, where $w_{i}$ refers to dimension weight and $s_{i}$ indicates dimension score (0–5). Complete scoring instructions with evaluation guidelines are outlined in Appendix A.

#### 2.2.2. Terminology Standardization

To achieve a common understanding and uniformity, this analysis establishes a common understanding by defining the following terms:

Emotional Annotation: Assigning labels to segments of EEG data that reflect emotional states (through subject reports, observational behavioral measures, or experimental induction protocols). Emotional states may reflect categories such as happiness, stress, frustration or dimensional ratings such as valence or arousal.

Affective Monitoring: The ongoing process of monitoring and appraising emotional states over time using physiological signals, which includes measuring the emotional state, along with interpreting emotional dynamics during driving tasks.

Emotion Recognition: The computational challenge of automatically classifying or predicting emotional states from physiological data using machine learning or deep learning models, assessed in terms of classification accuracy of prediction performance using regression metrics.

Affective States: This term is used more broadly to refer to emotions, moods or feelings that may influence driver behavior, and includes ephemeral experiences of emotion as well as longer-lasting mood dispositions.

Cognitive States: These terms refer to mental processes and capacities including tasks related to cognitive load, attention, fatigue, and working memory, which impact driving performance directly.

## 3. Results

After exploring the selected databases, a comparison was conducted on two dimensions, construction characteristics and performance results, as reported in the literature. This dual approach enabled evaluation of the theoretical construction and its practical use.

### 3.1. Comparative Analysis of Dataset Construction

This section presents a comparison of dataset construction characteristics, including modality integration, scope of physiological monitoring, data accessibility levels, subject pool size, gender distribution, and age demographics.

#### 3.1.1. Dataset Modality

**Figure 3:**
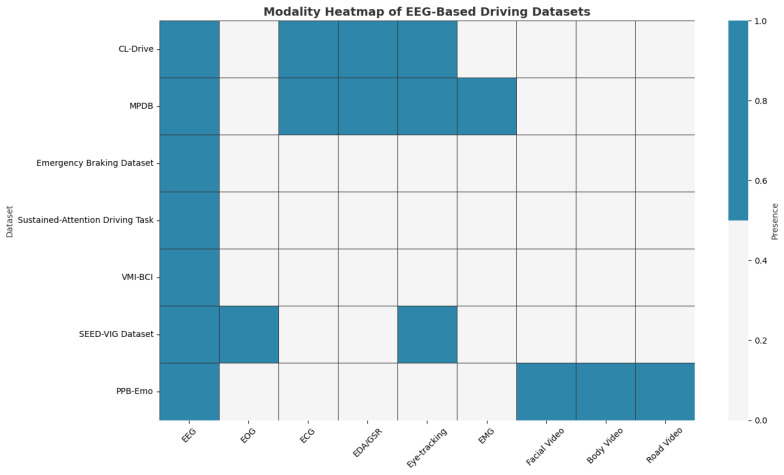
Modality integration heatmap across seven EEG-based driving datasets.

The chosen datasets include additional modalities aside from EEG. Figure 3 shows a visualization of modality integration across the seven EEG-based driving datasets. The x-axis distinguishes between modalities, including EEG, EOG (Electrooculography), ECG (Electrocardiography), EDA/GSR (Electrodermal Activity/Galvanic Skin Response), eye-tracking, and EMG (Electromyography). The y-axis represents the datasets. The shading of each cell indicates the presence or absence of a given modality within a dataset. EEG is present in all datasets, but auxiliary modalities such as EOG, ECG, and EDA/GSR are present only in a subset. Video modalities (Facial Video, Body Video, and Road Video) exist in only one dataset, whereas higher-resolution neuroimaging modalities capable of measuring cortical hemodynamics or magnetic fields (fNIRS (Functional Near-Infrared Spectroscopy), MEG (Magnetoencephalography), and fMRI (Functional Magnetic Resonance Imaging)) are absent from all datasets used in this research.

To clarify the auxiliary modalities illustrated in Figure 3, a short description of each signal type is provided in Table 3.

#### 3.1.2. Scope

The thematic distribution assessment, depicted in Table 4, indicates an inconsistency across driving EEG studies. Drowsiness detection represents the most frequently investigated theme, represented in two datasets and approached nearly twice as often as the other themes, which included cognitive load assessment, emergency braking intention, driving behaviour analysis, visual–motor imagery and emotion recognition, each appearing in only one dataset. As a result, the datasets investigated place a significantly larger emphasis on passive state monitoring (drowsiness detection) compared to decoding active behavior and decision-making.

#### 3.1.3. Dataset Accessibility

The majority, 71.4%, of the studied driving EEG datasets are openly accessible, while the remaining, 28.6%, are restricted, typically under an “available on request” model.

#### 3.1.4. Size and Gender Distribution

**Figure 4:**
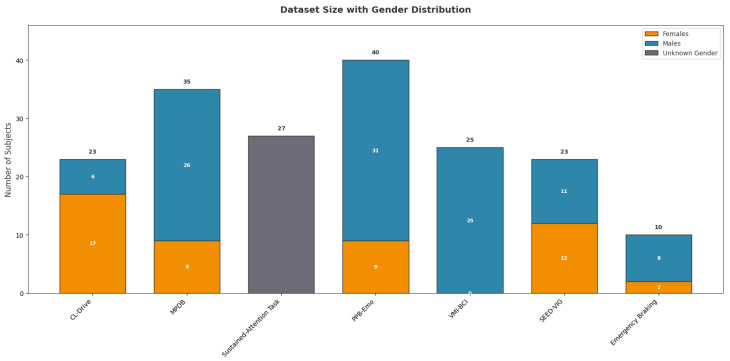
Dataset size and gender distribution.

Figure 4 illustrates the gender distribution across EEG datasets used in driving research. A marked gender imbalance is evident, with a strong male prevalence in most datasets. Exceptions include the SEED-VIG dataset, which is nearly balanced (12 females, 11 males), and the CL Drive dataset, which contains more females than males. The Sustained-Attention Task dataset presents metadata issues, as it did not indicate gender for its 27 subjects. Overall, the aggregate distribution (68.6% male, 31.4% female) demonstrates a systemic gender imbalance in these EEG datasets.

#### 3.1.5. Age Distribution

**Figure 5:**
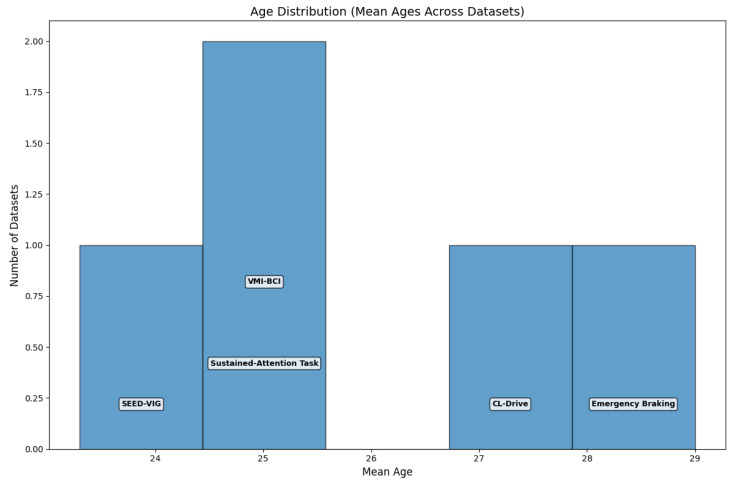
Age distribution (mean ages across datasets).

An examination of participant ages across the five datasets that reported this information (Figure 5) identifies a general focus on young adults, with mean ages concentrated around 25 years. Some datasets reported slightly higher mean ages (28–29 years), and one dataset reported a mean age of approximately 23 years.

#### 3.1.6. Quantitative Multidimensional Assessment

The quantitative application of the proposed scoring framework reveals significant differences in dataset quality and suitability for driver monitoring research, as shown in Table 5.

Analysis of the quantitative data reveals that MPDB achieves the highest composite score (4.05/5) among all datasets, due to its advantages across multiple dimensions, including demographic diversity, modality richness, and technical quality. Conversely, ecological validity is the weakest dimension across datasets (mean 2.1/5), as no dataset included data collected outside of a simulation. Accessibility achieved the highest mean score (mean = 4.3), reflecting positive trends in open data practices; however, demographic balance remained an issue (mean = 2.5), as most datasets showed significant age or gender bias. Direct comparisons of performance between datasets are invalid due to differing tasks and evaluation paradigms, as shown in Table 6. Nevertheless, qualitative analysis suggests that high-scoring datasets like MPDB and PPB-Emo likely enabled more complex model architectures; those with diverse modalities supported multimodal fusion approaches, and those with better technical quality facilitated more reproducible research.

### 3.2. Comparative Analysis of Model Performance

In this part, we will discuss how to evaluate model performance in a data analysis framework with three facets:

- Algorithm Performance: Assess models on neuro-physiological data.
- Emerging Techniques: Provide examples of recent advances.
- Transferability and Benchmarking: Address cross-dataset issues.

The goal is to select high-performing models, understand current shortcomings, and outline future directions while providing recommendations for model selection and generalizability.

#### 3.2.1. Algorithm Performance

The analysis of performance indicates clear advantages of specialized architectures and approaches in comparison to traditional methods. Deep learning models consistently outperformed traditional machine learning approaches with multimodal physiological data. The study cited in [1] employed the XGBoost algorithm, a gradient-boosted decision tree ensemble, and achieved 83.67% accuracy on the CL-Drive dataset by successfully fusing the features extracted from the EEG, ECG, EDA, and gaze modalities. In contrast, VGG-style CNNs in [1] utilized convolutional layers to learn hierarchical features from the same multimodal data input with 79.35% accuracy. In more complex tasks, the performance gap is even more pronounced: on MPDB, MMPNet [14] (an MMPNet architecture) achieved an absolute accuracy over linear discriminant analysis (LDA) of 27.5% (62.6% to 35.1%), which demonstrates LDA’s limitations in modeling nonlinear relationships between physiological measures.

Multimodal integration continuously resulted in performance increases across paradigms. For example, combining EEG with peripheral physiological signals (ECG, EDA) and gaze data results in greater than 20% accuracy improvements on CL-Drive [1] while MMPNet achieved 7% accuracy gains on MPDB when using multimodal instead of unimodal inputs [14]. While these gains are notable, they practically disappear in subject-independent validation. The leave-one-subject-out (LOSO) protocol showed common generalization issues: VGG’s accuracy dropped by 5% to 10% on CL-Drive when considering LOSO validation instead of standard validation [1], clearly demonstrating sensitivity to inter-subject differences [21].

Notably, task complexity comes with additional considerations for models. Transitioning from binary to ternary classification in CL-Drive resulted in a 15% loss in accuracy for VGG [1]. Task-specific hybrid architectures show greater resilience: CNN-LSTM models on VMI-BCI [17] achieved 85% accuracy where convolutional feature extraction and temporal modeling from a long short-term memory network architecture were used, producing 12% greater accuracy than SVM baselines.

The findings of the model performance comparison process are summarized in Table 6. The table provides the best algorithm for each dataset.

#### 3.2.2. Emerging Used Techniques

More recent methodological advances are specialized designs that respond to neurophysiological data characteristics. Because of the unique spatial, temporal, and spectral properties of neurophysiological data, these new specialized types of architecture present challenges for traditional models. Specialized designs provide a better fit for the non-linear characteristics of the dynamics formed in neurophysiological datasets, including a variety of distributions, and variability across datasets. A review of the literature associated with the selected datasets reveals a number of emerging specialized techniques:

Sequence modeling: The research conducted in [15,22] employs transformer networks, which utilize self-attention mechanisms by allowing the model to learn and incorporate long-range dependencies for EEG time series, providing a productive dimension of temporal space to assist long-term analysis in driver sustained attention analysis. The benefits of these networks are particularly relevant for sustained attention during long driving sessions, as they eliminate RNN structure and omit the limitations associated with RNNs, where it is hard, if not impossible, to maintain context over long durations. Furthermore, the overall estimation of workload in automated vehicles improved state-of-the-art accuracy by nearly 8 to 12% over conventional methods [22], allowing for the detection of attention lapses earlier, which could play a role in accidents, during driving states.

Graph-based learning: High-order relationships between classifying modalities in MPDB are modeled using hypergraph convolutional networks (MMPHGCN: Multi-Modal Physiological Hypergraph Convolutional Network) [23], which leverage hypergraph convolutional neural networks to model non-linear cross-signal interactions. This network was effective in modeling the complicated physiological interactions occurring during driving operations, including autonomic responses (EDA), muscle tone (EMG), and brain states (EEG). Representing multimodal dependencies as hyper edges reduces false positives in collision warning systems and produces 15 to 20% greater accuracy in driver intention detection compared to standard graph models [23].

Spatial hierarchy preservation: Capsule networks [5] retain the spatial relationships of EEG topographies during fatigue detection in SEED-VIG and they significantly outperformed standard CNNs when preserving neuroanatomical information. The hierarchical part–whole relationships encoded by capsule networks between clusters of electrodes allowed for better detection of fatigue signatures that existed spatially (frontal alpha increases and occipital beta decreases at the same time). In fact, capsule attention mechanisms increase accuracy in detecting fatigue by 7 to 9% and decrease false alerts by 12% [5], which is enough to increase reliability of overnight driving monitoring where standard CNNs cannot confidently differentiate between drowsy and normal relaxation patterns.

Representation learning: Attention mechanisms such as the feature masking in HATNet [24] and capsule attention [25] dynamically weight relevant EEG components, mimicking cognitive saliency so that the mechanisms can ignore less relevant neural noise. MIN2Net [15] uses metric learning to create discriminative embeddings for vigilance tasks, where similar high-dimensional EEG cognitive states are tightly clustered on compact manifolds. MIN2Net had 88.7% accuracy for emotion recognition, which is 5.2% greater than autoencoders [15] without compression, preserving inter-trial relationships, while the use of HATNet improved cognitive load classifications by 9.3% relative to using a baseline with just the raw EEG, by over-emphasizing features that are critical to the cognition process [24], enabling more granular cognitive workload measurements when tasks are cognitively complex, like parallel parking.

Data augmentation: Generative adversarial networks (GANs) [15] produce realistic EEG samples that can help alleviate the data scarcity challenge, which is especially useful for rare safety-critical scenarios. Additionally, GANs can generate synthetic versions of emergency responses without the risks of real road testing, effectively removing data collection barriers that are ethical. Strategic augmentation of EEG samples improves EEGNet’s AUC score from 0.64 to 0.94 in the prediction of intention to brake in [16]. This was particularly valuable, being 200 ms ahead of actual braking, which is a key safety time frame. The results showed AUC = 0.94/F1 = 0.65 for emergency vs. normal driving, and AUC = 0.91/F1 = 0.85 for emergency vs. normal braking [16], and importantly reduces the overall lag of prediction time by 300% versus existing approaches.

Fusion strategies: From basic feature concatenation to architectures that preserve spatiotemporal relationships, fusion strategies have changed over time. End-to-end models in CL-Drive [1], MPDB [14], and SEED-VIG [5] show that complementary dynamics are better captured by raw data integration using specialized deep learning blocks than by late fusion techniques. They overcome synchronization issues between rapid neural signals (ms (millisecond) scale) and slower physiological responses (s (second) scale) by processing temporal gaze patterns and spectral EEG features together in unified architectures. When compared to unimodal inputs, multimodal fusion increased accuracy by over 20% in CL-Drive [1] and 7% in MPDB [14]. This allowed for a comprehensive evaluation of driver state in situations where single-modality systems are unable to function properly, such as when talking while changing lanes.

Self-supervised continual learning: An emerging method for dealing with non-stationary streaming data is self-supervised continual learning. This is demonstrated by the framework SSOCL (Bi-Level Self-Supervised Continual Learning), which bootstraps learning using a pretrained model on the PPB-EMO dataset [26]. Then, using a dynamic memory buffer and pseudo labeling, it continuously adjusts to new, unlabeled subjects, attaining a high generalization accuracy of 71.78% on the DEAP dataset [26]. This illustrates how well it can recognize emotions in dynamic environments with robustness and label efficiency.

Cross-Domain Spatiotemporal Modeling Advances: Recent advancements in spatiotemporal modeling of EEG from similar fields suggest promising models that could be used for driver state monitoring. STEADYNet [27] uses a lightweight convolutional architecture to detect dementia and achieves very high accuracy (up to 99.65%) using minimal computational complexity (446,963 parameters), which suggests it could be implemented in real time. Similarly, STRFLNet [28] utilizes dynamic-static graph topologies with continuous graph ordinary differential equations for emotion recognition and is able to capture both stable and transient brain connectivity. These models in particular demonstrate the promise of spatiotemporal-based modeling that could be adapted to more effectively capture the complex and dynamic nature of driver states while achieving computational efficiency for in-vehicle implementations.

#### 3.2.3. Transferability and Benchmarking

Studies involving cross-dataset transfer and cross-subject generalization consistently show significant performance degradation as a result of distribution shifts brought on by demographic differences, environmental variations, and device heterogeneity [16,17]. Although reported accuracy drops vary considerably, in difficult settings they frequently surpass 15%. Model performance is highly sensitive to the features of the dataset because of

- Differences in signal resolution (4 vs. 59 EEG channels);
- Labeling granularity;
- Inconsistencies in the acquisition protocol.

There are still no unified benchmark studies that evaluate models like EEGNet, VGG, and SVM under uniform training and evaluation protocols, despite the fact that these models have been independently tested on datasets like CL-Drive, MPDB, Emergency Braking, and VMI-BCI [1,14,16,17]. Meaningful conclusions regarding cross-dataset robustness are impeded by this gap.

Comparative evaluations of model performance and dataset construction taken together show a great deal of technological innovation. But they also reveal important, recurring trends in demographic biases and methodological shortcomings. These results are summarized in the discussion that follows in order to assess the obstacles that presently limit the generalizability and real-world applicability of EEG-based driving research.

## 4. Discussion

This section discusses the limitations identified by the proposed analysis and suggests recommendations to address them.

### 4.1. Limitations

Following the proposed methodology, many key issues require in-depth discussion. This study reveals a significant disparity between the current state of EEG-based driving research and its practical applicability. Despite considerable technical progress, six primary limitations hinder real-world deployment:

1. Age Bias in Participant Demographics: Across all datasets, there is a persistent trend toward early adulthood. Six of the seven cohorts have mean ages ranging from 22 to 28 years, with only two datasets extending past 35 years (MPDB: 20–60 years, PPB-Emo: 19–58 years). Age bias towards the young adult sample (mean age: 25 years) results in poor performance for older drivers. The study in [29] estimated a 22–27% drop in accuracy when models were applied to populations not represented in the training data. This is problematic because of age-related changes in neural processing speed and cognitive workload [30].
2. Dependence on Simulated Environments: Existing datasets are exclusively derived from simulated or imagined driving conditions. These setups fail to capture the complexities and unpredictability of real-world driving, including environmental noise, abrupt maneuvers, and infrastructure irregularities. Empirical evidence suggests that simulator-based evaluations may overestimate system performance by 20 to 30% [31], thereby limiting external validity. In addition, driving in real time also creates several EEG challenges specific to driving, including motion artifacts from vibration, electromagnetic interference with vehicle electronics, and more complex attentional demands that will induce changes in spectral patterns and topographical distributions [32]. In addition, new methodologies, such as graph learning [33], can model non-stationary brain connectivity under genuine distractions, and self-supervised learning can train robust representations against noisy real-world data, all bridging the simulation-to-reality gap [34].
3. Absence of Affective State Monitoring: While affective states have firmly established roles in understanding behaviors and accident causation, there is only one dataset that includes direct measures of driver emotion (PPB-Emo). Drowsiness is typically covered, but many other emotional dimensions (anxiety, stress, boredom, fear, excitement) that could be informative of risky behavior remain unmonitored. The limited emotional coverage does not enable the models to recognize important states like frustration or road rage, since those signals have unique neural signatures that differ from the more common drowsiness.
4. Gender Bias Toward Male Participants: The datasets exhibit a clear male bias (68.6% male). Gender imbalances create models that do not capture neural representations specific to male or female participants. This is shown in the existing research evidencing considerable sex/gender differences in cognitive abilities and neural processing that will modify the characteristics of the EEG signals [35].
5. Limitations in Data Accessibility: Some datasets can be accessed freely using repositories like Figshare or GitHub, though a substantial portion (Emergency Braking, VMI-BCI) are provided only by the author upon request. This creates an inequitable research ecosystem whereby validation and progress rely on researcher goodwill. Furthermore, such restrictions prevent replication, secondary analysis, and the creation of multimodal large-scale benchmarks.
6. Lack of Cross-Dataset Validation: The field lacks vigorous evidence for generalizable models. The common method of training and testing on single datasets leads to overfitting to specific populations, protocols, and hardware characteristics. Additionally, heterogeneity among the evaluation protocols, preprocessing pipelines, and model architectures remains a barrier towards making cross-dataset performance comparisons or model generalization.

### 4.2. Recommendations

These combined limitations severely affect the real-world efficacy of EEG driver monitoring systems. To address these challenges and bridge the gap between research and real-world application, a major priority should be to apply more robust and generalizable study designs. This analysis suggests a multi-faceted approach:

Enhancing Demographic and Ecological Generalizability: To address this, it is critical to add participants from a wider age (18–80 years) and gender range to facilitate balanced participation. Also, gathering data in operational vehicles, starting with instrumented testing environments, will deliver the base data needed to build models that generalize well across the whole driving population in real-world settings. Future research should focus not only on increasing the size and diversity of the participant pool, but also on methods to reduce bias by implementing stratified sampling with explicit, researcher-defined quotas for demographic variables such as age categories (18–30, 31–50, 51–70, 70+) and gender. This needs to be followed up with stratified model evaluation to investigate and measure whether there are differences in performance between subgroups, as this analysis indicates that accuracy decreased by as much as 27% for underrepresented groups [29,30]. Future research should develop methods of data collection that progressively transition from instrumented testing environments to research opportunities in real-road conditions to better capture the full variety of driver populations and driving conditions.

Incorporating Affective Measures: To simulate affective state variability, it is necessary to include tasks that induce frustration, stress (complex decision making under time constraints), and euphoria (open highway driving). In these instances, there must also be a clear synchrony between multiple sensory recordings (EMG, speech analysis, fNIRS, and videos of facial expressions). Future research protocols should apply multimodal ground truth that synchronizes self-report, behavioral observation (facial/vocal analysis) and physiological coherence in ecologically valid driving situations to anchor emotional labels. Section 2.2 provides initial principles for a terminology framework, but developing universal instructions that can be applied across research contexts remains a community challenge due to the subjective nature of emotion.

Implementing a Graduated Validation Framework: To address technical difficulties associated with cross-dataset validation, such as domain shift resulting from differences in EEG devices, mismatches in feature distributions across devices, and calibration inconsistencies, this study proposes a structured pipeline that progresses from laboratory to simulator, then closed-track and finally open-road scenarios for progressively realistic validation. Importantly, this structured approach requires the use of domain adaptation techniques, like domain-adversarial training [36], at each step to learn features that are invariant to the device. It also requires the use of continuous calibration methods to align feature distributions among different acquisition systems. This graduated approach, with consistent exposure to increasing realism and consistent actions to address shifts in distributions, provides quantifiable measures of performance decline at each stage of exposure and enables more reliable transfer of models to real-world use.

Bias Mitigation Strategies: To mitigate demographic biases and their consequences on performance, we recommend the below methods.

Domain Adaptation: Domain-adversarial training [36] learns invariant features across demographic groups, minimizing distribution shifts between the majority and underrepresented populations.

Fair Representation Learning: Algorithms that obscure protected attributes while preserving task-relevant information [37] are applied, thereby enforcing demographic parity without requiring explicit labels during inference.

Data Augmentation: Generative approaches like EEGGAN-Net [38] synthesize EEG samples for underrepresented groups, balancing distributions without additional data collection.

Transfer Learning: Pre-training on balanced clinical/BCI datasets before fine-tuning on driving data reduces reliance on biased driving datasets.

Stratified Validation: Demographic-stratified cross-validation ensures realistic performance estimates across all subgroups.

Adopting Advanced Spatiotemporal Architectures: Building on recent cross-domain advances, future driver monitoring systems should leverage advanced spatiotemporal modeling techniques. For instance, the continuous graph propagation in STRFLNet [28] can mitigate the over-smoothing typical of traditional GCNs when modeling long-range spatial dependencies during driving. Similarly, STEADYNet [27] maintains high performance with minimal computational overhead, a design principle well-suited to vehicular embedded systems. Consequently, future data collection must ensure sufficient spatial coverage (electrode density) and temporal resolution to support such sophisticated models, while capturing multimodal interactions for robust fusion strategies.

## 5. Conclusions

EEG-based driver monitoring represents a remarkable transition in automotive safety, by evaluating the cognitive and affective states of drivers. However, real-world systems remain difficult to achieve due to inconsistencies in the databases used. In this context, this study conducts the first multidimensional benchmark of seven EEG driving datasets, evaluating them in terms of dataset construction and model performance. Our systematic review approach, preregistered and following PRISMA guidelines, provides comprehensive insights while acknowledging the inherent limitations of synthesizing heterogeneous literature. The analysis confirms that while the field has made significant technical advancements through deep learning models, with accuracy improvements ranging from 7 to 27%, these advancements are constrained by foundational limitations. These include widespread demographic bias towards young male participants that can lead to accuracy drops of up to 27% for older drivers, a simulation–reality gap where controlled conditions may overestimate true performance by 20 to 30%, and a narrow focus on drowsiness that neglects other critical affective states implicated in a substantial portion of accidents. In addition, the restricted accessibility of many datasets continues to slow independent validation and rapid progress.

Therefore, this study recommends that future work should prioritize the collection of more inclusive, age-diverse data in valid experimental settings, progressing from simulations to closed-track and ultimately open-road environments. Experimental protocols should be redesigned to incorporate a wider range of emotions. Finally, adopting open science principles with standardized sharing practices is crucial for building robust, generalizable benchmarks.

## Figures and Tables

**Table 1:** Characteristics of selected publicly available EEG driving datasets.

Dataset	Year	Subjects	Age	Gender	EEG Channels	Access	Openness
MPDB	2024	35	20–60	26M, 9F	59	Figshare	★★★
SEED-VIG	2024	23	23.3 ± 1.4	11M, 12F	17	Figshare	★★★
CL-Drive	2023	21	26.9	6M, 17F	4	Github	★★★
Emergency Braking	2023	10	22–36	8M, 2F	28	Author request	★★
PPB-Emo	2022	40	19–58	31M, 9F	32	Figshare	★★★
VMI-BCI	2021	25	25 ± 1	All M	18	Author request	★★
Sustained-Attention	2019	27	22–28	-	32	Figshare	★★★

Note: All datasets were collected in simulated driving environments. Openness scoring: ★★★ = High Openness (publicly available with open license), ★★ = Moderate Openness (available on request). All links were accessed on 17 June 2025.

**Table 2:** Multidimensional scoring rubric.

Dimension	Wt	5	3	1	0
Demographic	0.20	Wide age and gender	Mod. diversity	Limited	Severe bias
Ecological	0.25	Real road	High-fidelity sim	Basic sim	Passive
Modality	0.15	≥3 aux	2 aux	1 aux	EEG-only
Annotation	0.15	Multi-modal cont.	Multi-discrete	Basic labels	No/binary
Accessibility	0.10	CC-BY direct	On request	Restricted	Unavailable
Technical	0.15	Full docs, >30	Good docs, 15–30	Limited, 10–15	Poor, <10

**Table 3:** Overview of modalities in driving datasets.

Modality	Captured Signal
EEG (Electroencephalography)	Electrical brain activity from the scalp
EOG (Electrooculography)	Corneo-retinal potential (eye movements)
ECG (Electrocardiography)	Electrical activity of the heart
EDA/GSR (Electrodermal Activity/ Galvanic Skin Response)	Skin conductance (sweat gland activity)
Eye-tracking	Gaze direction and fixation points
EMG (Electromyography)	Muscle activity (surface electrodes)

**Table 4:** Scope of driving datasets.

Dataset	Scope
MPDB	Driving Behavior Analysis
CL-Drive	Cognitive Load Assessment
Emergency Braking	Emergency Braking Intention
PPB-Emo	Emotion Recognition
VMI-BCI	Visual–Motor Imagery
Sustained-Attention	Drowsiness Detection
SEED-VIG	Drowsiness Detection

Note: All links were accessed on 17 June 2025.

**Table 5:** Dataset multidimensional scores and rankings.

Dataset	Demographic	Ecological	Modality	Annotation	Access.	Technical	Comp.	Rank
MPDB	4.2	3.5	4.8	3.2	5.0	4.5	4.05	1
PPB-Emo	3.8	2.0	4.2	4.5	5.0	4.0	3.58	2
CL-Drive	2.5	3.0	4.5	3.0	5.0	3.0	3.33	3
SEED-VIG	3.0	1.0	3.0	3.0	5.0	3.5	2.83	4
Sustained-Attention	1.0	3.0	2.0	2.0	5.0	3.5	2.65	5
Emergency Braking	2.0	3.0	2.0	2.0	3.0	2.0	2.35	6
VMI-BCI	1.0	2.0	2.0	2.0	3.0	3.0	2.15	7

**Table 6:** Best-performing models by dataset.

Dataset	Model	Performance	Key Strength
CL-Drive	XGBoost (multimodal)	83.67% Acc	Feature fusion effectiveness
MPDB	MMPNet (multimodal)	62.6% Acc	Multimodal integration
Sustained-Attention	CNN	95.2% Acc	Spatial–temporal feature extraction
SEED-VIG	DNNSN (LSTM-based)	PCC 0.8237	Temporal dependency modeling
Emergency Braking	EEGNet	AUC 0.94	High temporal precision
VMI-BCI	SVM-EOG hybrid	85% Acc	Multi-signal integration
PPB-Emo	MDERNet (dual-branch DL)	High accuracy (study-specific)	Mid-level multimodal feature fusion

Note: Performance metrics are reported from their respective original papers and are task-dependent. They are intended to summarize the state of the art for each dataset specifically and are not directly comparable between tasks due to task dependent evaluation paradigms and choices in metric definition.

## Data Availability

Not applicable.

## Supplementary Materials

The following supporting information can be downloaded at https://www.mdpi.com/article/10.3390/s25247426/s1: PRISMA Checklist for the systematic review of EEG driving datasets.

## Appendix A. Complete Multidimensional Scoring Guidelines

### Appendix A.1. Scoring Methodology

All datasets were rated on six dimensions using the 0–5 point scale as specified in Table 2. Scoring was determined using objective criteria and published documentation associated with the dataset.

### Appendix A.2. Scoring Criteria for the Specific Dimension

#### Appendix A.2.1. Demographic Diversity

- Five points: Age range > 40 years and gender-balanced distribution (45–55% for males and females);
- Three points: Age range between 25 and 40 years and moderate gender distribution (30–70% for males and females);
- One point: Age range narrow (<10 years) and severe gender imbalance (>80% single gender);
- Zero points: A single demographic group or no demographic information.

#### Appendix A.2.2. Ecological Validity

- Five points: Real-road driving with naturalistic conditions;
- Three points: High-fidelity driving simulator with interactive scenarios;
- One point: Basic simulation or simplified driving tasks;
- Zero points: Passive video viewing or non-driving contexts.

#### Appendix A.2.3. Modality Richness

- Five points: ≥3 auxiliary modalities (EOG, ECG, EDA, eye-tracking, etc.);
- Three points: 2 auxiliary modalities;
- **One point**: 1 auxiliary modality;
- Zero points: EEG-only recording.

#### Appendix A.2.4. Annotation Quality

- Five points: Multi-modal continuous annotation (continuous ratings + behavioral coding);
- Three points: Multiple discrete labels across sessions;
- One point: Basic task labels only;
- Zero points: No labels or binary classification only.

#### Appendix A.2.5. Accessibility

- Five points: CC-BY license, direct download;
- Three points: Available on request with academic use allowed;
- One point: Restricted access or unclear licensing;
- Zero points: Not available to the public.

#### Appendix A.2.6. Technical Quality

- Five points: Full documentation, >30 subjects;
- Three points: Good documentation, 15–30 subjects;
- One point: Limited documentation, 10–15 subjects;
- Zero points: Poor documentation, <10 subjects.

### Appendix A.3. Dataset-Specific Score Justifications

#### Appendix A.3.1. MPDB (Composite: 4.05)

- Demographic: 4.2: Wide age range (20–60) but gender imbalance (74% male);
- Ecological: 3.5: High-fidelity simulation but not real-road;
- Modality: 4.8: EEG + EOG + EDA + gaze + vehicle data (five modalities);
- Annotation: 3.2: Multiple discrete labels for driving behavior;
- Accessibility: 5.0: CC-BY, direct download via Figshare;
- Technical: 4.5: Comprehensive documentation, 35 subjects.

#### Appendix A.3.2. PPB-Emo (Composite: 3.58)

- Demographic: 3.8: Good age range (19–58) but gender imbalance (78% male);
- Ecological: 2.0: Basic simulated driving tasks;
- Modality: 4.2: EEG + physiological + behavioral data (3+ modalities);
- Annotation: 4.5: Multi-modal emotion annotation;
- Accessibility: 5.0: CC-BY, direct download via Figshare;
- Technical: 4.0: Good documentation, 40 subjects.

#### Appendix A.3.3. CL-Drive (Composite: 3.33)

- Demographic: 2.5: Narrow age (26.9 mean), female-biased (81% female);
- Ecological: 3.0: High-fidelity simulation;
- Modality: 4.5: EEG + ECG + EDA + gaze (four modalities);
- Annotation: 3.0: Cognitive load labels across tasks;
- Accessibility: 5.0: CC-BY, direct download via GitHub (commit 44b0334, accessed October 2024);
- Technical: 3.0: Limited documentation, 21 subjects.

#### Appendix A.3.4. SEED-VIG (Composite: 2.83)

- Demographic: 3.0: Narrow age (23.3 ± 1.4), nearly balanced gender;
- Ecological: 1.0: Passive video viewing only;
- Modality: 3.0: EEG + forehead EOG (two modalities);
- Annotation: 3.0: Vigilance state labels;
- Accessibility: 5.0: CC-BY, direct download via Figshare;
- Technical: 3.5: Good documentation, 23 subjects.

#### Appendix A.3.5. Sustained-Attention (Composite: 2.65)

- Demographic: 1.0: Narrow age (22–28), no gender information;
- Ecological: 3.0: High-fidelity driving simulation;
- Modality: 2.0: EEG-only recording;
- Annotation: 2.0: Basic attention state labels;
- Accessibility: 5.0: CC-BY, direct download via Figshare;
- Technical: 3.5: Good documentation, 27 subjects.

#### Appendix A.3.6. Emergency Braking (Composite: 2.35)

- Demographic: 2.0: Moderate age range (22–36), gender imbalance (80% male);
- Ecological: 3.0: High-fidelity simulation;
- Modality: 2.0: EEG-only recording;
- Annotation: 2.0: Basic braking intention labels;
- Accessibility: 3.0: Available on request only;
- Technical: 2.0: Limited documentation, 10 subjects.

#### Appendix A.3.7. VMI-BCI (Composite: 2.15)

- Demographic: 1.0: Narrow age (25 ± 1), all-male participants;
- Ecological: 2.0: Basic simulation tasks;
- Modality: 2.0: EEG-only recording;
- Annotation: 2.0: Basic motor imagery labels;
- Accessibility: 3.0: Available on request only;
- Technical: 3.0: Limited documentation, 25 subjects.

**Table A1:** Details of excluded datasets and justification.

Dataset Name	Primary Reason for Exclusion
EEG Driver Fatigue Detection	Non-peer-reviewed publication (Kaggle source only)
Sleepy Driver EEG Brainwave Data	Non-peer-reviewed publication + Insufficient participant count (number of subjects = 4)
EEG Dataset Recorded In A Car Simulator	Non-peer-reviewed publication (Kaggle source only)
Multimodal Cognitive Load Classification Dataset	Non-peer-reviewed publication (Kaggle source only)
Cognitive load during driving dataset	Restricted data access
Driving Physiological and Vehicle Data Multimodal Fusion Dataset (DPV-MFD)	Restricted data access

Note: All links were accessed on 17 June 2025.
