# Supplementary material for: A Multidimensional Benchmark of Public EEG Datasets for Driver State Monitoring in Brain–Computer Interfaces
Source: Sensors (Basel). 2025 Dec 6;25(24):7426. doi: 10.3390/s25247426 (PMC12737006; doi:10.3390/s25247426)
Supplement: Supplementary file 1 [file sensors-25-07426-s001.zip › sensors-3989798-supplementary.pdf]

PRISMA 2020 Checklist

Section and Topic	Item #	Checklist item	Location where item is reported
**TITLE**			
Title	1	Identify the report as a systematic review.	**Page 1, Title** **Relevant text:** "A Multidimensional Benchmark of Public EEG Datasets for Driver State Monitoring in Brain-Computer Interfaces"
**ABSTRACT**			
Abstract	2	See the PRISMA 2020 for Abstracts checklist.	**Page 1, Abstract** **Relevant text:** The entire abstract summarizes the objective, methods, results, and conclusions of the systematic review.
**INTRODUCTION**			
Rationale	3	Describe the rationale for the review in the context of existing knowledge.	**Page 2, Paragraph 2** **Relevant text:** "Many research studies have examined cognitive states in driving but contributions have often been limited by issues related to datasets such as restricted data access, practical uses of datasets and

PRISMA 2020 Checklist

Section and Topic	Item #	Checklist item	Location where item is reported
			differences in methodology."
Objectives	4	Provide an explicit statement of the objective(s) or question(s) the review addresses.	**Page 3, Paragraph 1** **Relevant text:** "To address this issue, this study provides for the first time... a structured and quantitative benchmark analysis of publicly available EEG datasets relevant to vehicle driving contexts."
**METHODS**			
Eligibility criteria	5	Specify the inclusion and exclusion criteria for the review and how studies were grouped for the syntheses.	**Page 4, Section 2.1.1** **Relevant text:** "The original selection of datasets is made based on three guiding principles: it uses EEG signals, involves driving related scenarios, and is in the public domain." Additional refinements (peer-reviewed, recent, participant thresholds) are

Section and Topic	Item #	Checklist item	Location where item is reported
			detailed on Page 5.
Information sources	6	Specify all databases, registers, websites, organisations, reference lists and other sources searched or consulted to identify studies. Specify the date when each source was last searched or consulted.	**Page 5, Section 2.1.2** **Relevant text:** "The comprehensive literature and dataset search was conducted across many information sources including: IEEE Xplore, ScienceDirect, arXiv, FigShare, Kaggle, GitHub, Google Scholar, and PhysioNet."
Search strategy	7	Present the full search strategies for all databases, registers and websites, including any filters and limits used.	**Page 5, Section 2.1.2** **Relevant text:** "The key search terms were 'EEG', 'driving', 'dataset', 'brain-computer interface', 'drowsiness detection'. The search included peer reviewed articles and datasets publicly accessible online."
Selection process	8	Specify the methods used to decide whether a study met the inclusion criteria of the review, including how many reviewers screened each record and each report retrieved, whether they worked independently, and if applicable, details of automation tools used in the process.	**Location: OSF, "Screening" ->**

Section and Topic	Item #	Checklist item	Location where item is reported
			**"Screening reliability" Relevant Text:** "A single reviewer conducted all screening stages. To ensure consistency... a pilot screening phase was conducted... The excluded records at the full-text stage were independently cross-checked by a second reviewer to validate the exclusion decisions."
Data collection process	9	Specify the methods used to collect data from reports, including how many reviewers collected data from each report, whether they worked independently, any processes for obtaining or confirming data from study investigators, and if applicable, details of automation tools used in the process.	**Location: OSF, "Extraction" -> "Extraction reliability" Relevant Text:** "A single primary extractor performed the data extraction. To ensure reliability: A pre-piloted, structured data extraction form was used... A second reviewer performed a verification check."

Section and Topic	Item #	Checklist item	Location where item is reported
Data items	10a	List and define all outcomes for which data were sought. Specify whether all results that were compatible with each outcome domain in each study were sought (e.g. for all measures, time points, analyses), and if not, the methods used to decide which results to collect.	**Page 5, Section 2.2** **Relevant text:** "It consists of: the number of subjects, the EEG channels, the type of task and the types of labels. Furthermore, all machine learning/deep learning models with performance data issued from the citation analysis are recorded."
	10b	List and define all other variables for which data were sought (e.g. participant and intervention characteristics, funding sources). Describe any assumptions made about any missing or unclear information.	**Page 6, Table 1** **Relevant text:** Table 1 extracts and defines variables including Year, Subjects, Age, Gender, EEG Channels, Access, and Environment.
Study risk of bias assessment	11	Specify the methods used to assess risk of bias in the included studies, including details of the tool(s) used, how many reviewers assessed each study and whether they worked independently, and if applicable, details of automation tools used in the process.	**Page 5, Section 2.1.1 (Refinements)** **Relevant text:** The review assessed quality indirectly via criteria such as "Peer-reviewed

Section and Topic	Item #	Checklist item	Location where item is reported
			publication", "Complete metadata", and "strict quality standards require documentation on applied signal preprocessing, artifact removal, and annotation reliability". **Location: OSF, "Synthesis and Quality Assessment" -> "Quality assessment"** **Relevant Text:** "We developed a custom quality assessment framework specific to EEG datasets, evaluating: Methodological Rigor... Data Completeness... Representativeness... Accessibility & Reproducibility."
Effect measures	12	Specify for each outcome the effect measure(s) (e.g. risk ratio, mean difference) used in the synthesis or presentation of results.	**Page 9-12, Section 3.2** **Relevant text:** The synthesis uses

Section and Topic	Item #	Checklist item	Location where item is reported
			performance metrics common in ML/BCI research, as shown in Table 4 and the text: "accuracy" (e.g., 83.67%), "PCC" (Pearson Correlation Coefficient), "AUC" (Area Under the Curve), and "F1" score.
Synthesis methods	13a	Describe the processes used to decide which studies were eligible for each synthesis (e.g. tabulating the study intervention characteristics and comparing against the planned groups for each synthesis (item #5)).	**Page 5, Section 2.2 Relevant text:** The synthesis is based on the 7 selected datasets. Analysis is grouped by "construction characteristics" (3.1) and "model performance" (3.2).
	13b	Describe any methods required to prepare the data for presentation or synthesis, such as handling of missing summary statistics, or data conversions.	**Page 6, Table 1 & Openness Scoring Relevant text:** Metadata was tabulated (Table 1). A formalized openness scoring system was created to categorize data accessibility.
	13c	Describe any methods used to tabulate or visually display results of individual studies and syntheses.	**Throughout Results**

Section and Topic	Item #	Checklist item	Location where item is reported
			**Relevant text:** Results are presented using tables (Table 1, 2, 3, 4) and figures (Figure 3, 4, 5) for comparative visualization.
	13d	Describe any methods used to synthesize results and provide a rationale for the choice(s). If meta-analysis was performed, describe the model(s), method(s) to identify the presence and extent of statistical heterogeneity, and software package(s) used.	**Page 9-12, Section 3.2** **Relevant text:** A narrative synthesis was conducted. The rationale is to compare "trends in model performance" across datasets. The text synthesizes results by comparing algorithm performance, emerging techniques, and transferability.
	13e	Describe any methods used to explore possible causes of heterogeneity among study results (e.g. subgroup analysis, meta-regression).	**Page 12, Section 3.2.3** **Relevant text:** Heterogeneity is explored by discussing causes for performance

Section and Topic	Item #	Checklist item	Location where item is reported
			differences, such as "demographic differences, environmental variations, and device heterogeneity", "Differences in signal resolution", and "Labeling granularity".
	13f	Describe any sensitivity analyses conducted to assess robustness of the synthesized results.	**Page 9, Section 3.2.1** **Relevant text:** The use of "leave-one-subject-out (LOSO) protocol" is discussed as a method that "showed common generalization issues", acting as a form of sensitivity analysis on subject dependence.
Reporting bias assessment	14	Describe any methods used to assess risk of bias due to missing results in a synthesis (arising from reporting biases).	**Page 6, "Openness Score"** **Relevant text:** The assessment of data accessibility ("High", "Moderate", "Low" Openness) directly

Section and Topic	Item #	Checklist item	Location where item is reported
			addresses reporting bias related to data availability.
Certainty assessment	15	Describe any methods used to assess certainty (or confidence) in the body of evidence for an outcome.	**Implicit in Methodology** **Relevant text:** While not using a formal GRADE-like system, certainty is assessed through the stringent inclusion criteria (peer-review, complete metadata, recent collection) which ensure a baseline quality and relevance of the included evidence.
**RESULTS**			
Study selection	16a	Describe the results of the search and selection process, from the number of records identified in the search to the number of studies included in the review, ideally using a flow diagram.	**Page 4-5, Section 2.1** **Relevant text:** "The search identified 13 datasets. After going through a further iterative selection process, 7 datasets are identified as candidates for inclusion."
	16b	Cite studies that might appear to meet the inclusion criteria, but which were excluded, and explain why they were excluded.	**Page 4-5, Section**

Section and Topic	Item #	Checklist item	Location where item is reported
			**2.1.1 Relevant text:** Examples of excluded datasets are given (e.g., from [2], [3], [4]) with reasons: "the authors do not indicate how to access their dataset", "this dataset too is not available", "the dataset was only described... and is not openly accessible".
Study characteristics	17	Cite each included study and present its characteristics.	**Page 6, Table 1 Relevant text:** Table 1 presents the characteristics of the 7 included datasets (MPDB, SEED-VIG, etc.), with citations provided in the Reference list.
Risk of bias in studies	18	Present assessments of risk of bias for each included study.	**Page 8, 12-13, Sections 3.1.4, 3.1.5 & 4.1 Relevant text:** Bias is presented as findings: "a marked

Section and Topic	Item #	Checklist item	Location where item is reported
			gender imbalance" (Fig 4), "a general focus on young adult ages" (Fig 5), and discussed as limitations "Age Bias", "Gender Bias".
Results of individual studies	19	For all outcomes, present, for each study: (a) summary statistics for each group (where appropriate) and (b) an effect estimate and its precision (e.g. confidence/credible interval), ideally using structured tables or plots.	**Page 10, Table 4** **Relevant text:** Table 4, "Best Performing Models by Dataset", presents the key outcome (model performance) for each included dataset, including the model name and its performance metric (Accuracy, AUC, etc.).
Results of syntheses	20a	For each synthesis, briefly summarise the characteristics and risk of bias among contributing studies.	**Page 6-8, Section 3.1** **Relevant text:** Sections 3.1.1-3.1.5 synthesize dataset characteristics (modality, scope, accessibility, demographics) which encompass risk of bias elements.

Section and Topic	Item #	Checklist item	Location where item is reported
	20b	Present results of all statistical syntheses conducted. If meta-analysis was done, present for each the summary estimate and its precision (e.g. confidence/credible interval) and measures of statistical heterogeneity. If comparing groups, describe the direction of the effect.	**Page 9-12, Section 3.2** **Relevant text:** The narrative synthesis presents results, e.g., "Deep learning models consistently outperformed traditional machine learning approaches", "multimodal integration... resulted in performance increases", and provides specific accuracy gains.
	20c	Present results of all investigations of possible causes of heterogeneity among study results.	**Page 12, Section 3.2.3** **Relevant text:** "Studies involving cross dataset transfer... consistently show significant performance degradation as a result of distribution shifts brought on by demographic differences, environmental

Section and Topic	Item #	Checklist item	Location where item is reported
			variations, and device heterogeneity."
	20d	Present results of all sensitivity analyses conducted to assess the robustness of the synthesized results.	**Page 9, Section 3.2.1** **Relevant text:** The impact of different validation methods is presented as a finding: "The leave-one-subject-out (LOSO) protocol showed common generalization issues: VGG's accuracy dropped by 5% to 10%."
Reporting biases	21	Present assessments of risk of bias due to missing results (arising from reporting biases) for each synthesis assessed.	**Page 8, Section 3.1.3** **Relevant text:** "The majority, 71.4%, of the studied driving EEG datasets are openly accessible, while the remaining, 28.6%, are restricted under 'available on request' models."
Certainty of evidence	22	Present assessments of certainty (or confidence) in the body of evidence for each outcome assessed.	**Page 12-13, Section 4.1 (Limitations)** **Relevant text:** The discussion on

Section and Topic	Item #	Checklist item	Location where item is reported
			limitations (e.g., "Dependence on Simulated Environments", "Lack of Cross-Dataset Validation") qualifies the certainty and real-world applicability of the evidence.
**DISCUSSION**			
Discussion	23a	Provide a general interpretation of the results in the context of other evidence.	**Page 12, Section 4** **Relevant text:** "this study reveals a significant disparity between the current state of EEG based driving research and its practical applicability."
	23b	Discuss any limitations of the evidence included in the review.	**Page 12-13, Section 4.1** **Relevant text:** Six primary limitations are discussed in detail: "Age Bias", "Dependence on Simulated Environments", "Absence of Affective State Monitoring",

Section and Topic	Item #	Checklist item	Location where item is reported
			etc.
	23c	Discuss any limitations of the review processes used.	**Page 5, Section 2.1.1 (Refinements) Relevant text:** The inclusion criteria themselves (e.g., "Recent collection", "Elevated participant thresholds") are stated and define the scope and potential limitations of the review.
	23d	Discuss implications of the results for practice, policy, and future research.	**Page 13-14, Section 4.2 (Recommendations) Relevant text:** Specific recommendations are provided for "Enhancing Demographic and Ecological Generalizability", "Incorporating Affective Measures", and "Implementing a Graduated Validation Framework".
**OTHER INFORMATION**			
Registration and protocol	24a	Provide registration information for the review, including register name and registration number, or state that the review was not registered.	**Open Science**

Section and Topic	Item #	Checklist item	Location where item is reported
			**Framework (OSF)** **Relevant text:** "This review was conducted in accordance with the PRISMA (Preferred Reporting Items for Systematic Reviews and Meta-Analyses) guidelines." (From the OSF "Review Methods" section). The review is registered on the OSF platform.
	24b	Indicate where the review protocol can be accessed, or state that a protocol was not prepared.	**Open Science Framework (OSF) Registration** **Relevant text:** The entire OSF registration page serves as the review protocol. It is publicly accessible at the provided OSF link.
	24c	Describe and explain any amendments to information provided at registration or in the protocol.	**Open Science Framework (OSF), "Review Methods" section** **Relevant text:** "This registration is an

Section and Topic	Item #	Checklist item	Location where item is reported
			update to document the finalized methodology prior to manuscript submission."
Support	25	Describe sources of financial or non-financial support for the review, and the role of the funders or sponsors in the review.	**Open Science Framework (OSF), "Funding" section** **Relevant text:** "This research received no specific grant from any funding agency in the public, commercial, or not-for-profit sectors."
Competing interests	26	Declare any competing interests of review authors.	**Open Science Framework (OSF), "Conflicts of interest" and "Overlapping authorships" sections** **Relevant text:** "The authors declare no conflicts of interest." ... "The authors of this review are not creators of any of the datasets included in the analysis."
Availability of data, code and	27	Report which of the following are publicly available and where they can be found: template data collection forms; data extracted from included studies; data used for all analyses; analytic code; any other materials used in the review.	**Open Science**

PRISMA 2020 Checklist

Section and Topic	Item #	Checklist item	Location where item is reported
other materials			**Framework (OSF), "Data management and sharing" sections (under Screening and Extraction) Relevant text (from Extraction section):** "The final, cleaned dataset containing all extracted entities will be shared as part of this registration. File Format: LaTeX table source file (.tex). Repository: Open Science Framework (OSF)."

From: Page MJ, McKenzie JE, Bossuyt PM, Boutron I, Hoffmann TC, Mulrow CD, et al. The PRISMA 2020 statement: an updated guideline for reporting systematic reviews. BMJ 2021;372:n71. doi: 10.1136/bmj.n71. This work is licensed under CC BY 4.0. To view a copy of this license, visit https://creativecommons.org/licenses/by/4.0/
